# Public Health Attention for Physical Activity

**Published:** 2006-12-15

**Authors:** Kristy Hansen

**Affiliations:** National Society of Physical Activity Practitioners in Public Health, Nutrition, Physical Activity, and Obesity Prevention Program, Texas Department of State Health Services, Austin, Tex

## To the Editor:

I would like to thank Sarah Levin Martin and Tammy Vehige for bringing national attention to the need for professional capacity in physical activity public health programs in their letter, "Establishing Public Health Benchmarks for Physical Activity Programs," in the July 2006 issue *of Preventing Chronic Disease *(1). Qualified physical activity and public health specialists provide critical technical assistance for state- and community-based interventions, and public health benchmarks for physical activity programs provide a sound framework for program development at the state health department level.

One immediate outcome of the benchmarks from the Centers for Disease Control and Prevention (CDC) is the formation of professional core competencies by the National Society of Physical Activity Practitioners in Public Health (NSPAPPH). These standards are specific to physical activity and public health practice, and NSPAPPH is the first professional organization dedicated to growing the capacity of physical activity practitioners in public health. Those who work in this emerging field come from various academic backgrounds and professions, including health promotion and education, public health, exercise science and exercise physiology, and physical education. Until now there has not been a set of professional standards specific to the field. The NSPAPPH Core Competencies and Training Committee used CDC's public health benchmarks for physical activity programs as a framework to develop core competencies, which are already being used across the United States to hire physical activity staff at state health departments, plan public health physical activity interventions, and inform institutions that train these professionals.

NSPAPPH intends to elevate physical activity in public health practice at national, state, and local levels through professional development. The public health benchmarks for physical activity programs have created a standard of practice for practitioners to use for years to come.
